# Pressure-induced electronic phase separation of magnetism and superconductivity in CrAs

**DOI:** 10.1038/srep13788

**Published:** 2015-09-08

**Authors:** Rustem Khasanov, Zurab Guguchia, Ilya Eremin, Hubertus Luetkens, Alex Amato, Pabitra K. Biswas, Christian Rüegg, Michael A. Susner, Athena S. Sefat, Nikolai D. Zhigadlo, Elvezio Morenzoni

**Affiliations:** 1 Laboratory for Muon Spin Spectroscopy, Paul Scherrer Institute, CH-5232 Villigen PSI, Switzerland; 2 Institut für Theoretische Physik III, Ruhr-Universität Bochum, D-44801 Bochum, Germany; 3 Kazan (Volga region) Federal University, 420008 Kazan, Russia; 4 Laboratory for Neutron Scattering and Imaging, Paul Scherrer Institute, CH-5232 Villigen PSI, Switzerland; 5 Department of Quantum Matter Physics, University of Geneva, CH-1211 Geneva, Switzerland; 6 Materials Science and Technology Division, Oak Ridge National Laboratory, Oak Ridge, TN 37831-6114, USA; 7 Solid State Physics Laboratory, ETH Zurich, 8093 Zurich, Switzerland

## Abstract

The recent discovery of pressure (*p*) induced superconductivity in the binary helimagnet CrAs has raised questions on how superconductivity emerges from the magnetic state and on the mechanism of the superconducting pairing. In the present work the suppression of magnetism and the occurrence of superconductivity in CrAs were studied by means of muon spin rotation. The magnetism remains bulk up to *p* 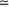 3.5 kbar while its volume fraction gradually decreases with increasing pressure until it vanishes at *p* 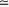 7 kbar. At 3.5 kbar superconductivity abruptly appears with its maximum *T*_*c*_ 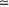 1.2 K which decreases upon increasing the pressure. In the intermediate pressure region (3.5 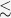 *p* 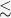 7 kbar) the superconducting and the magnetic volume fractions are spatially phase separated and compete for phase volume. Our results indicate that the less conductive magnetic phase provides additional carriers (doping) to the superconducting parts of the CrAs sample thus leading to an increase of the transition temperature (*T*_*c*_) and of the superfluid density (*ρ*_*s*_). A scaling of *ρ*_s_ with 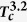 as well as the phase separation between magnetism and superconductivity point to a conventional mechanism of the Cooper-pairing in CrAs.

The pressure-induced superconductivity in the binary helimagnet CrAs has recently attracted much attention^1^,^2^,^3^,^4^,^5^. At ambient pressure CrAs is characterized by a relatively high Néel temperature 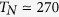 K^6^,^7^,^8^. *T*_*N*_ decreases approximately by a factor of three for pressures (*p*) approaching 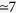 kbar, above which the magnetism completely disappears^1^,^2^,^3^. On the other hand superconductivity sets in for pressures exceeding 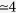 kbar thus revealing a range of 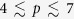 kbar where superconductivity and magnetism coexist.

The close proximity of superconductivity to magnetism, the similarity of the phase diagram of CrAs with that of some Fe-based superconductors, as well as the absence of the coherent Hebel-Slichter peak in the nuclear relaxation rate 1/*T*_1_*T* made the authors of Refs 1, 2, 3, 5 to suggest an unconventional pairing mechanism. It should be noted, however, that the similarity of the phase diagram does not necessarily requires a similar mechanism of Cooper-pairing. The Hebel-Slichter peak can also be suppressed in conventional *s*—wave superconductors. This is *e.g.* the case for superconductors in the strong coupling limit^9^, for superconductors having a spread of *T*_*c*_ over the sample, or for slight gap anisotropies^10^. Whether a coherence peak is present at all in the archetypical two-gap superconductor MgB_2_ is still subject of discussion^11^,^12^. One needs, therefore, a more detailed investigation of the superconducting response of CrAs as well as an understanding on how superconductivity emerges from a compound being initially in a strong magnetic state.

In this paper we report on muon spin rotation (*μ*SR) studies of the magnetic and the superconducting properties of CrAs. We first discuss separately the magnetic and the superconducting responses as a function of pressure, and concentrate later on the issue of coexistence between magnetism and superconductivity.

## Results

### Magnetism in CrAs

The magnetic response of CrAs powder samples was studied by zero field (ZF) and weak transverse field (wTF) *μ*SR experiments. In the following we discuss the *μ*SR data for three different pressure regions.

**Figure 1:**
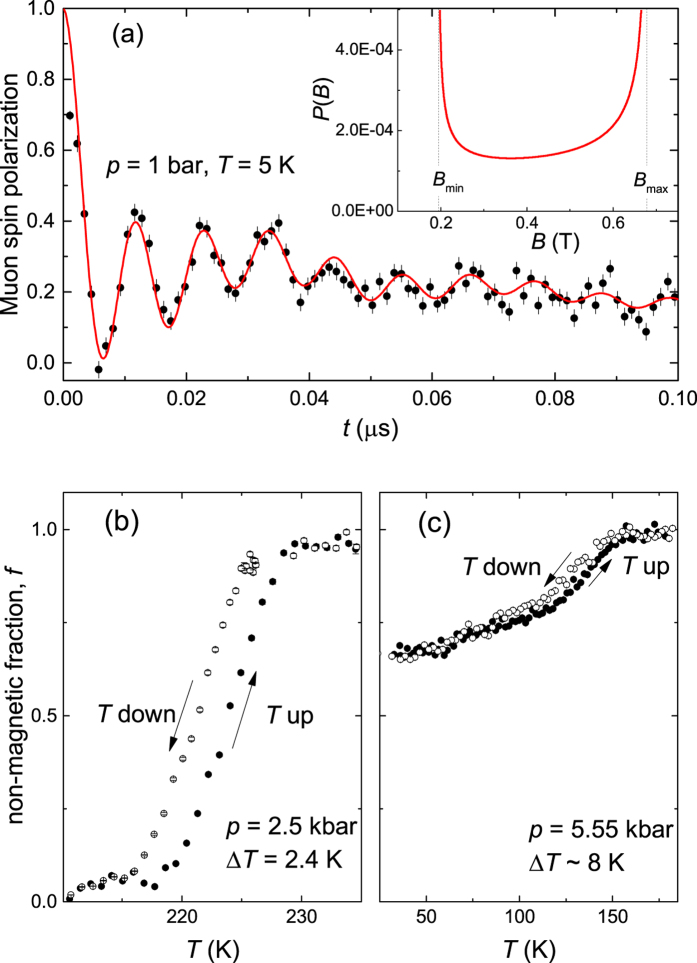
Representative ZF and wTF *μ*SR data. (**a**) ZF *μ*SR time-spectra of CrAs measured at *T* = 5 K and *p* = 1 bar. The solid line is a fit according to the theoretical field distribution caused by incommensurate helimagnetic order shown in the inset [see the Supplemental materials for details]. The minimum (*B*_*min*_) and the maximum (*B*_*max*_) cutoff fields are represented by vertical dashed lines. (**b**) and (**c**) depict the temperature evolution of the non-magnetic volume fraction *f* of CrAs obtained in the wTF *μ*SR measurements at *p* = 2.5 and 5.55 kbar, respectively. Closed and open symbols correspond to the experimental data obtained with increasing and decreasing temperature (the sweeping rate is 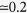 K/min, 5 minutes per data point). The clear hysteresis is indicative of a first order magnetic transition.

In the low-pressure region, 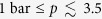 kbar, spontaneous muon spin precession is clearly seen in the ZF *μ*SR time spectra (see Fig. 1a) thus confirming that long range magnetic order is established below *T*_*N*_. The oscillating part of the signal is accurately described by a field distribution characterized by a minimum (*B*_*min*_) and a maximum (*B*_*max*_) cutoff field (see the inset in Fig. 1a), which is consistent with the observation of helimagnetic incommensurate magnetic order^4^,^5^,^6^,^7^,^8^. The relatively high values of the cutoff fields (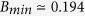 T and 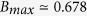 T at *p* = 1 bar) are in agreement with the large moments 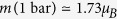 as obtained by means of neutron powder diffraction^4^. The wTF *μ*SR experiments performed at ambient pressure and at *p* = 2.5 kbar show relatively sharp transitions to the magnetic state and prove that the magnetism occupies close to 100% of the sample volume (see Fig. 1b and Fig. Sup 3 in the Supplemental material). The hysteresis in *T*_*N*_ signifies a first order magnetic phase transition.

In the intermediate pressure region (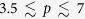 kbar) the cutoff fields, which are proportional to the ordered moment, decrease continuously and reach at 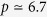 kbar 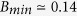 T and 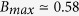 T (see Fig. Sup 1 in the Supplemental materials). This is consistent with a decrease of the ordered magnetic moment to 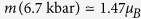. The wTF *μ*SR experiments reveal that the low temperature value of the non-magnetic fraction *f* gradually increases with increasing pressure (see Fig. 1b,c and Fig. Sup 3 in the Supplemental materials). Therefore in the intermediate pressure region the sample is separated into a magnetically ordered phase and a non-magnetic phase. The hysteresis in *T*_*N*_ confirms that the magnetic transition remains of first order at all pressures (see Fig. 1c).

For pressures above 7 kbar the ZF *μ*SR experiments prove the absence of any type of magnetic order as exemplified by the weakly damped wTF *μ*SR time spectra.

### Superconductivity in CrAs

The superconducting response of CrAs was studied in transverse field (TF) *μ*SR experiments (applied field *μ*_0_*H* = 30 mT). From the experimental data we have extracted the magnetic penetration depth *λ*, which is related to the superfluid density *ρ*_*s*_ in terms of *ρ*_*s*_ = *n*_*s*_/m^*^ ∝ *λ*^−2^ (*n*_*s*_ charge carrier concentration and m^*^ carrier effective mass). The magnetic penetration depth 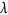 was determined from the Gaussian muon-spin depolarization rate *σ*_*sc*_(*T*) ∝ *λ*^−2^(*T*), which reflects the second moment of the magnetic field distribution in the superconductor in the mixed state^13^. *σ*_*sc*_ is related to *λ* via 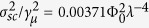
14 (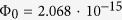 Wb is the magnetic flux quantum, and 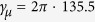 MHz/T is the muon gyromagnetic ratio).

**Figure 2:**
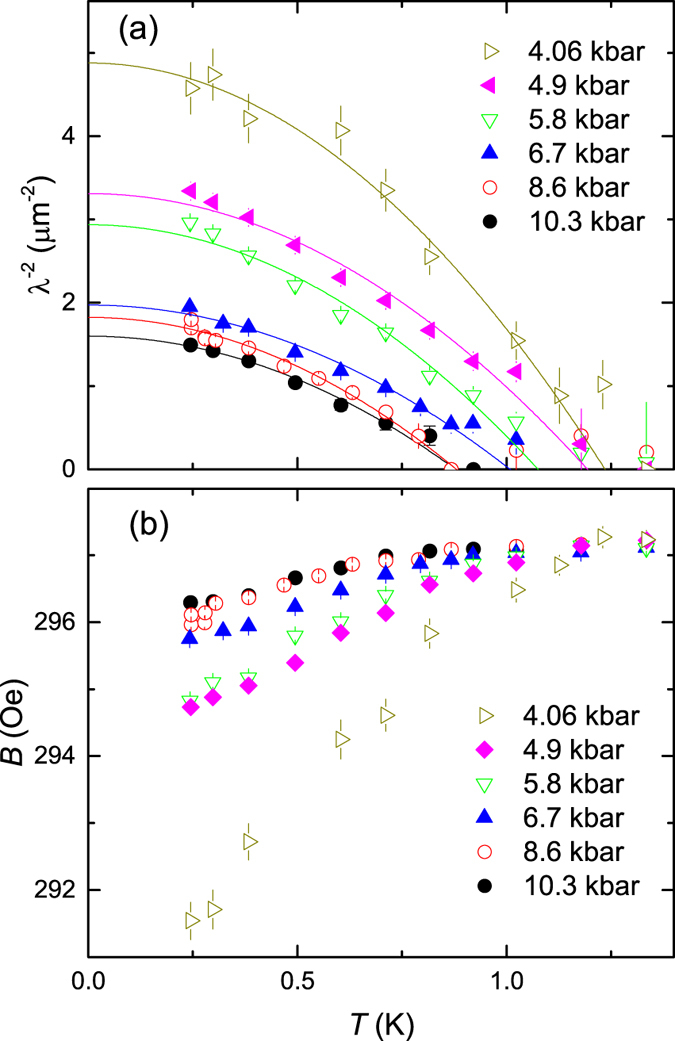
The superfluid density and the diamagnetic shift at various pressures. (**a**) Temperature evolution of the inverse squared magnetic penetration depth 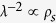 and (**b**) the internal field *B* obtained from the fit of 30 mT TF *μ*SR data measured at *p* = 4.06, 4.9, 5.8, 6.7, 8.6, and 10.3 kbar. Solid lines in (**a**) are power law fits 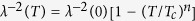 with a common exponent *n* = 1.95(3) for all data sets.

**Figure 3:**
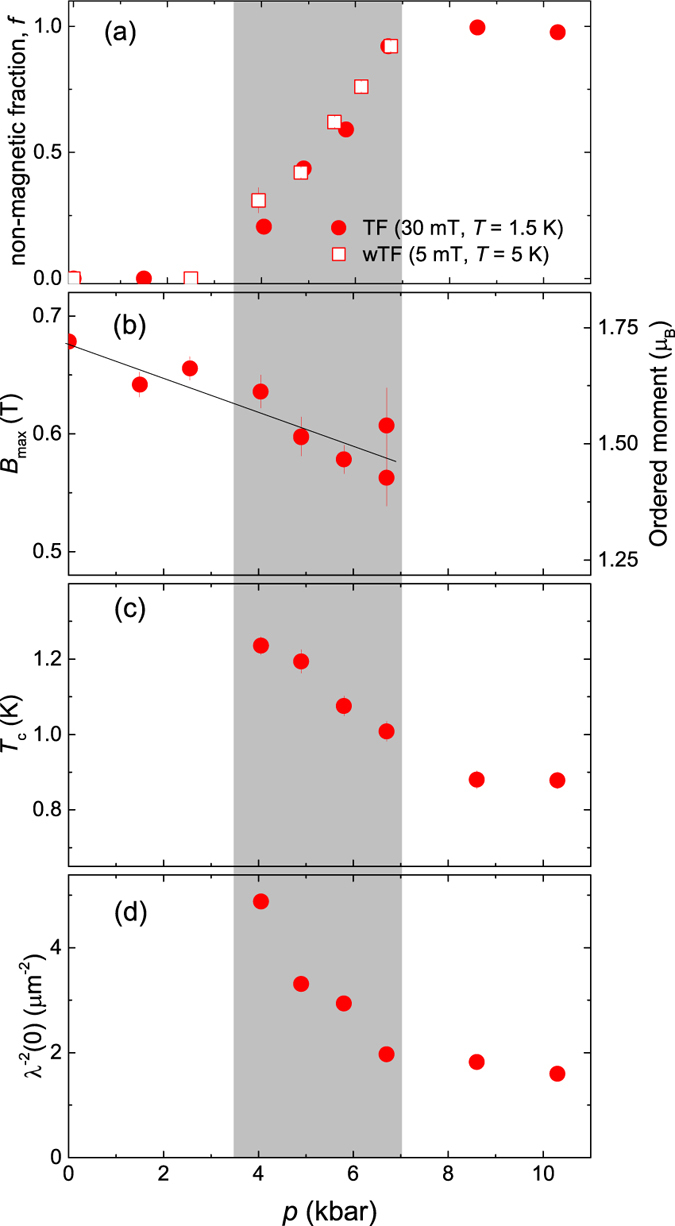
Temperature-pressure phase diagram. (**a**) Pressure dependence of the non-magnetic volume fraction *f*; (**b**) maximum cutoff field *B*_*max*_, which is proportional to the ordered moment 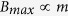; (**c**) superconducting transition temperature *T*_*c*_; and (**d**) the zero-temperature value of the inverse squared magnetic penetration depth 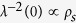. The grey area represents the pressure region where magnetism and superconductivity coexist. The solid line in (**b**) is a linear fit with 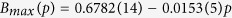 (see the Supplemental materials).

The measured *λ*^−2^(*T*) and the internal field *B*(*T*) of CrAs for *p* = 4.06, 4.9, 5.8, 6.7, 8.6 and 10.3 kbar are shown in Fig. 2a,b. Note that *λ*^−2^ and *B* were derived from the fraction of the sample remaining in the non-magnetic state down to the lowest temperature (see Fig. 3a). Due to the strongly damped signal in the magnetic phase one is unable to measure any superconducting response in the magnetic fraction of the sample. We believe, however that superconductivity in CrAs cannot emerge in the magnetically ordered parts for two following reasons. First, Wu *et al.*^1^ have shown that the low-temperature diamagnetic susceptibility (*χ*_*dia*_) of CrAs is nearly zero for pressures 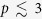 kbar, increases linearly in the range 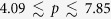 and reaches its maximum value, close to the ideal *χ*_*dia*_ = −1/4*π*, for pressures exceeding 7.85 kbar. It follows almost exactly the pressure dependence of the non-magnetic fraction *f* as observed in our wTF and TF *μ*SR experiments (see Fig. 3a). Second, the large magnetic moment and its weak reduction as a function of pressure (see Fig. 3b) require the separation of CrAs in superconducting and magnetic domains. This is *e.g.* the case for the so-called ‘245’ family of Fe-based superconductors^15^,^16^, which is characterized by the high value of both, magnetic moment (~3*μ*_*B*_) and Néel temperature (*T*_*N*_ ~ 500 K)^17^,^18^,^19^,^20^. Note that within the full pressure range studied here the value of the ordered magnetic moment in CrAs is only a factor of two smaller than that in ‘245’ superconductors.

The absence of experimental points below 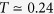 K prevents us from drawing any conclusion about the possible gap symmetry in CrAs based on the *λ*^−2^(*T*) data. Therefore, they were fitted to a power law 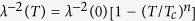 with the common exponent *n* = 1.95(3) for all data sets. Values for the superconducting transition temperature *T*_*c*_ and the inverse squared zero-temperature magnetic penetration depth *λ*^−2^(0) obtained from these fits are plotted in Fig. 3c,d.

### Interplay between magnetism and superconductivity

Figure 3 summarizes our results on the magnetism and superconductivity in CrAs as a function of pressure. CrAs remains purely magnetic up to 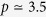 kbar. Above this pressure and up to 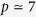 kbar both, magnetic and superconducting responses are clearly detected in a set of ZF, wTF, and TF *μ*SR experiments. CrAs is phase separated into volumes where long range magnetic order is established below the Néel temperature *T*_*N*_ and into non-magnetic volumes becoming superconducting below the critical temperature *T*_*c*_. It is interesting to note that, besides the competition for the volume, there is no evidence for a competition between the magnetic and superconducting order parameter in CrAs. This is in contrast to various Fe-based and cuprate superconductors where it is generally observed. Indeed, the ordered magnetic moment stays almost *constant*, by changing less than 15% from 1.73*μ*_*B*_ at *p* = 1 bar to 1.47*μ*_*B*_ at 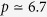 kbar, see Fig. 3b. *T*_*N*_, in their turn, evolves smoothly with pressure without showing any pronounced features at 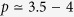 kbar, *i.e.* where the non-magnetic phase starts to develop (see Refs 1, 2, 3,5).

Figure 3a,d demonstrate that the maximum value of the superfluid density 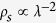 is observed at the low pressure side of the phase separated region *i.e.* in the region where the non-magnetic volume fraction *f* is the smallest. With further increasing *f*, the superfluid density decreases until it saturates when 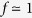. By neglecting the pressure effect on the charge carrier mass m^*^, the superfluid density is simply proportional to the carrier concentration *ρ*_*s*_ ∝ *n*_*s*_. We may assume, therefore, that within the phase separated region carriers from the ‘less conductive’ magnetically ordered parts of the sample can be supplied to the ‘more conductive’ non-magnetic parts, which become superconducting at low temperatures. The effect of supplying additional carriers, which can be considered as “doping”, is expected to be the strongest if the magnetic volume fraction exceeds substantially the paramagnetic one (*f* 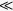 1), while it should decrease and even vanishes completely for *f* approaching 1. Figure 3a,d imply that this is exactly the case for CrAs. Effectively, the non-magnetic volume fraction *f anticorrelates* with the superfluid density *ρ*_*s*_.

### Correlation between *T*_*c*_ and *λ* ^−2^

**Figure 4:**
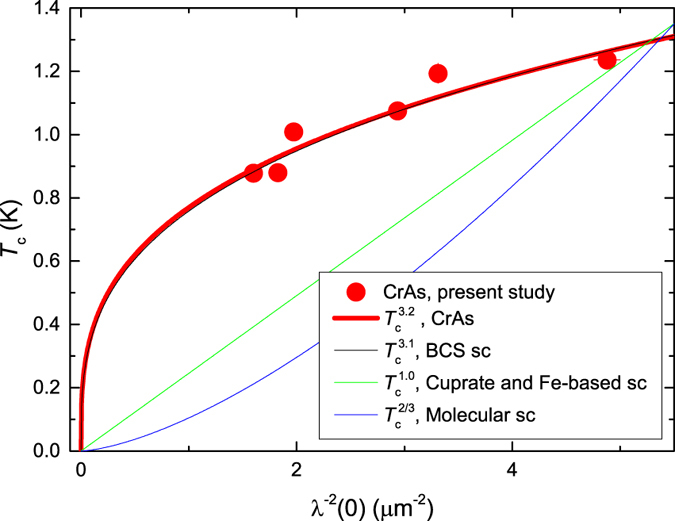
Correlation between *T*_*c*_ and *λ*^−2^(0). Superconducting critical temperature *T*_*c*_ versus inverse squared zero-temperature magnetic penetration depth *λ*^−2^(0) of CrAs. The red line is 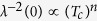 fit to CrAs data with the exponent *n* = 3.2(2). The black, green and blue lines are empirical relations for some phonon mediated BCS superconductors (*n* = 3.1, Ref. 28), cuprate and Fe-Based high-temperature superconductors (*n* = 1, Refs 21, 22, 23, 24, 25, 26) and molecular superconductors (*n* = 2/3, Ref. 27), respectively.

Figure 3c,d show that *T*_*c*_ and *λ*^−2^(0) have similar pressure dependences, which could point to a possible correlation between these quantities. The famous “Uemura line” establishes a linear relation between *T*_*c*_ and *λ*^−2^(0) for various families of underdoped cuprate high-temperature superconductors^21^,^22^. A similar linear relation was observed in recently discovered Fe-based superconductors^23^,^24^,^25^,^26^. In molecular superconductors *λ*^−2^(0) was found to be proportional to 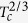 27, while in some phonon mediated BCS superconductors 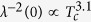 28. Figure 4 shows that in CrAs *λ*^−2^(0) the data scales as 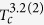 thus suggesting that superconductivity in CrAs is most probably BCS like and is mediated by phonons.

A further indication of conventional electron-phonon coupling in CrAs comes from the observed macroscopic phase separation of the magnetic and the superconducting phases. Following Ref. 29, the relative phase difference (*θ*) of the superconducting order parameter between different parts of Fermi surface or Fermi surface sheets may lead either to stabilization of microscopic coexistence of the magnetic and superconducting phases or drive both to repel each other. This happens because the staggered magnetic moment (**M**) plays in a superconductor the role of an intrinsic Josephson coupling with the free energy term 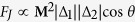 (Δ_1_, Δ_2_ are the superconducting order parameters at different parts of the Fermi surface or Fermi surface sheets). If the superconducting order is a conventional one, (*i.e.* there is no internal phase change, *θ* = 0), this term increases the total energy, thus making both phases unlikely to coexist. On the contrary, if the phases are opposite such that *θ* = *π* the Josephson coupling term in the free energy is negative. As a result both the superconducting and the magnetic phases like to coexist. This explains why the magnetic and superconducting orders do coexist microscopically in some unconventional superconductors like ferropnictides, electron-doped cuprates, and heavy-fermion systems where the order parameter has an internal phase shift. In CrAs, however, the phase diagram points towards an isotropic *s*—wave symmetry of the superconducting order parameter driven by electron-phonon interaction.

## Conclusions

To conclude, the magnetic and the superconducting properties of CrAs as a function of pressure were studied by means of muon spin rotation. The bulk magnetism exists up to 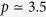 kbar, while the purely non-magnetic state develops for pressures above 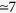 kbar. In the intermediate pressure region (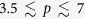 kbar) the magnetic phase volume decreases continuously and superconductivity develops in parts of the sample remaining non-magnetic down to the lowest temperatures. Both, the superconducting transition temperature *T*_*c*_ and the zero-temperature superfluid density *ρ*_*s*_(0) decrease with increasing pressure in the intermediate pressure region and saturate for *p* exceeding 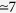 kbar *i.e.* in the region where magnetism is completely suppressed.

Our results suggest that the pressure-induced transition of CrAs from a magnetic to a superconducting state is characterized by a separation in macroscopic size magnetic and superconducting volumes. The less conductive magnetic phase provides additional carriers (doping) to the superconducting parts of CrAs. This would naturally explain the substantial increase of both, the transition temperature *T*_*c*_ (from 0.9 K to 1.2 K) and the superfluid density *ρ*_*s*_(0) (up to 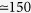
%), in the phase coexistence region. The superfluid density was found to scale with *T*_*c*_ as 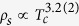, which, together with the clear phase separation between magnetism and superconductivity, points towards a conventional mechanism of the Cooper-pairing in CrAs.

## Methods

### Sample preparation

Two type of polycrystalline CrAs samples were used during our studies. The first type of sample was prepared by means of high-pressure synthesis. Overall details of the sample cell assembly and high-pressure synthesis process can be found in Ref. 30. The mixture of Cr (99.9%) and As (99.99%) powders in a molar ratio 1:1 was enclosed in a boron nitride (BN) crucible and placed inside a pyrophylite cube with a graphite heater. In a typical run, the sample was compressed to 15 kbar at room temperature. While keeping pressure constant, the temperature was ramped up to 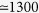 °C in 3 h, held there for a period of 9 h, and then cooled down to the room temperature in 3 h. Afterwards, the pressure was released and the sample removed. On two such synthesized samples the ZF and wTF *μ*SR experiments under ambient pressure were conducted.

The second type of polycristalline CrAs samples was synthesized by solid state reaction as described in^31^. The samples obtained by this method were used in ZF and wTF studies under ambient pressure and for all experimental studies under the pressure.

### Pressure Cell

The pressure was generated in a piston-cylinder type of cell made of CuBe alloy, which is especially designed to perform muon-spin rotation experiments under pressure^32^. As a pressure transmitting medium 7373 Daphne oil was used. The pressure was measured *in situ* by monitoring the pressure shift of the superconducting transition temperature of In. The maximum safely reachable pressures at *T* = 300 and 3 K are 14 and 11 kbar, respectively^32^.

### Muon-spin rotation (*μ*SR)

*μ*SR measurements at zero field (ZF) and field applied transverse to the initial muon-spin polarization were performed at the *π*M3 and *μ*E1 beamlines (Paul Scherrer Institute, Villigen, Switzerland), by using the GPS and GPD spectrometers, respectively. At the GPS spectrometer, equipped with a continuous flow ^4^He cryostat, ZF and 3 mT weak transverse field (wTF) experiments at ambient pressure and down to temperatures 1.6 K were carried out. At the GPD spectrometer, equipped with an Oxford sorption pumped ^3^He cryostat (base temperature ~0.24 K) and continuous flow ^4^He cryostat (base temperature 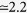 K), the ZF, 5 mT wTF, and 30 mT transverse field (TF) *μ*SR experiments under pressure up to ~10.3 kbar were conducted. All ZF and TF experiments were performed by stabilizing the temperature prior to recording the muon-time spectra. In the wTF experiments under pressure the temperature was swept up and down with the rate 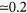 K/min. The data were collected continuously. Each muon-time spectra was recorded during approximately 5 minutes.

In a *μ*SR experiment nearly 100% spin-polarized muons are implanted into the sample one at a time. The positively charged muons thermalize at interstitial lattice sites, where they act as magnetic microprobes. The muon spin precesses about the local magnetic field *B* at the muon site with the Larmor frequency *ω*_*μ*_ = *γ*_*μ*_*B* (*γ*_*μ*_/2*π* = 135.5 MHz/T is the muon gyromagnetic ratio).

In pressure experiments a large fraction of the muons, roughly 50%, stop in the pressure cell walls adding a background contribution, which has to be separated from the sample signal in the data analysis. The detailed description of the data analysis procedure is given in the “Supplemental material” part.

## Additional Information

**How to cite this article**: Khasanov, R. *et al.* Pressure-induced electronic phase separation of magnetism and superconductivity in CrAs. *Sci. Rep.* **5**, 13788; doi: 10.1038/srep13788 (2015).

## Supplementary Material

Supplementary Information
