# Supplementary material for: Pressure-induced electronic phase separation of magnetism and superconductivity in CrAs
Source: Sci Rep. 2015 Sep 8;5:13788. doi: 10.1038/srep13788 (PMC4561900; doi:10.1038/srep13788)
Supplement: Supplementary Information [file srep13788-s1.pdf]

Supplemental materials for: "Pressure-induced electronic phase separation of magnetism and superconductivity in CrAs"

Rustem Khasanov[1,*], Zurab Guguchia[1], Ilya Eremin[2,3], Hubertus Luetkens[1], Alex Amato[1], Pbabitra K. Biswas[1], Christian Rüegg[4,5], Michael A. Susner[6], Athena S. Sefat[6], Nikolai D. Zhigadlo[7], and Elvezio Morenzoni[1]

[1]Laboratory for Muon Spin Spectroscopy, Paul Scherrer Institute, CH-5232 Villigen PSI, Switzerland
[2]Institut für Theoretische Physik III, Ruhr-Universität Bochum, D-44801 Bochum, Germany
[3]Kazan (Volga region) Federal University, 420008 Kazan, Russia
[4]Laboratory for Neutron Scattering and Imaging, Paul Scherrer Institute, CH-5232 Villigen PSI, Switzerland
[5]Department of Condensed Matter Physics, University of Geneva, CH-1211 Geneva, Switzerland
[6]Materials Science and Technology Division, Oak Ridge National Laboratory, Oak Ridge, TN 37831-6114, USA
[7]Solid State Physics Laboratory, ETH Zurich, 8093 Zurich, Switzerland
[*]rustem.khasanov@psi.ch

ABSTRACT

The Supplemental Material part contains information of the zero-field and the transverse field μSR data analysis procedure, as well as comparison of $T_c(p)$ obtained in the present study with the literature data.

Muon-spin rotation data analysis procedure

In pressure experiments a large fraction of the muons, roughly 50%, stop in the pressure cell walls. The fit function consists, therefore, of the "sample" and the background (pressure cell) contributions and is described as:

$$A(t) = A_s(0)P_s(t) + A_{pc}(0)P_{pc}(t). \quad (1)$$

Here $A_s(0)$ and $A_{pc}(0)$ are the initial asymmetries and $P_s(t)$ and $P_{pc}(t)$ are the muon-spin polarizations belonging to the sample and the pressure cell, respectively. The polarization of the pressure cell is generally studied in separated set of experiments (see *e.g. Ref.*[S1]).

ZF μSR experiments.

At ambient pressure CrAs is characterized by long-range helimagnetic order with a propagation vector $k_c = 0.3562(2)$ parallel to the $c-$axis and the magnetic moments lie in the ab plane.[S2] The ordered magnetic moment per Cr is $m \simeq 1.73\ \mu_B$.[S2] Due to the incommensurability of the magnetic structure, a continuous set of local fields is expected to be seen at each particular muon stopping site. It was shown that such a magnetic structure leads to a field distribution given by:[S1,S3,S4]

$$P(B) = \frac{2}{\pi}\frac{B}{\sqrt{(B^2 - B_{min}^2)(B_{max}^2 - B^2)}} \quad (2)$$

and is characterised by two peaks due to the minimum (B_{min}) and maximum(B_{max}) cutoff fields (see the inset in Fig. 1 in the main text). Considering only one muon stopping site, the ZF muon-spin polarization for a powder sample would follow the relation:[S4]

$$P_{ZF}(t) = \left[\frac{1}{3}e^{-\lambda_L t} + \frac{2}{3}e^{-\lambda_T t}\int P(B)\cos\gamma_\mu Bt\right]. \quad (3)$$

Here λ_T and λ_L are the transverse and the longitudinal exponential relaxation rates, respectively. The occurrence of 2/3 oscillating and 1/3 non–oscillating μSR signal fractions originates from the spatial averaging in powder samples, where 2/3 of the magnetic field components are perpendicular to the muon-spin and cause a precession, while the 1/3 longitudinal field components do not.

Figure 1 shows the dependence of the minimum B_{min} and the maximum B_{max} cutoff fields of CrAs on pressure. Points were obtained from the fit of ZF and wTF μSR data measured at $T \lesssim 5$ K. Both B_{min} and B_{max} decrease with increasing pressure. Following Ref.[S5] for a helical magnetic structure the upper and the lower cutoff fields should scale as $2m$ and m, respectively. Linear fits resulting in $\mathrm{d}B_{min}/\mathrm{d}p = -8.0(8)$ mT/kbar and $\mathrm{d}B_{max}/\mathrm{d}p = -15.3(5)$ mT/kbar thus confirm this statement.

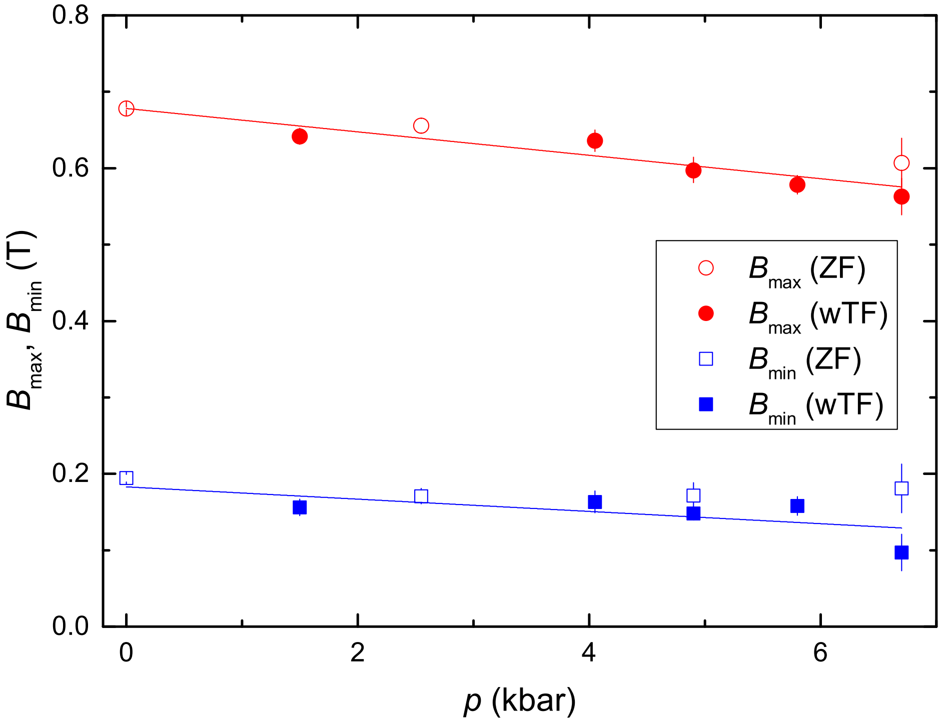

Figure 1. Pressure dependence of the minimum $B_{\rm min}$ and the maximum $B_{\rm max}$ cutoff fields of CrAs as obtained from the fit of ZF (open symbols) and wTF (closed symbols) μSR data for $T \lesssim 5$ K. The solid lines are linear fits with $B_{min}(p) = 0.1829(20) - 0.0080(8)p$ and $B_{max}(p) = 0.6782(14) - 0.0153(5)p$.

The decrease of B_{min} and B_{max} with increasing pressure implies a decrease of the ordered magnetic moment. By taking into account that the ambient pressure value of the ordered moment per Cr was found to be $\simeq 1.73\ \mu_B$[S2] our results would imply that with increasing pressure up to $p \simeq 6.7$ kbar Cr moments decrease down to $\simeq 1.47\ \mu_B$.

wTF μSR experiments.

μSR experiments under weak transverse field (wTF) applied perpendicular to the muon-spin polarization are a straightforward method to determine the onset of the magnetic transition and the magnetic volume fraction. In this case the contribution to the asymmetry from muons experiencing a vanishing internal spontaneous magnetisation can be accurately determined. Muons stopping in a non-magnetic environment produce long lived oscillations, which reflect the coherent muon precession around the external field B_{ex}. Muons stopping in magnetically ordered parts of the sample give rise to a more complex, distinguishable signal, reflecting the vector combination of internal and external fields. The random orientation of the grains in a powder sample leads to a broad distribution of precession frequencies.

The situation is substantially simplified for $B_{ex} \ll B_{int}$ (weak transverse field regime). In this case one can neglect the influence of B_{ex} on B_{int} and the fitting function becomes:

$$\begin{aligned} A(0)P(t) &= A_{nm}(0)\cos(\gamma_\mu B_{ex} t + \phi)\ e^{-\sigma_{nm}^2 t^2/2}. \\ &\quad + A_m(0)P_{ZF}(t) + A_{pc}(0)P_{pc}(t) \end{aligned} \tag{4}$$

Here $A_{nm}(0)$ and A_m are the initial non-magnetic and magnetic asymmetry, respectively, ϕ is the initial phase of the muon-spin ensemble, and σ_{nm} is the temperature independent Gaussian relaxation rate caused by nuclear moments. $P_{ZF}(t)$ represent the ZF magnetic polarization and is described by Eq. (3).

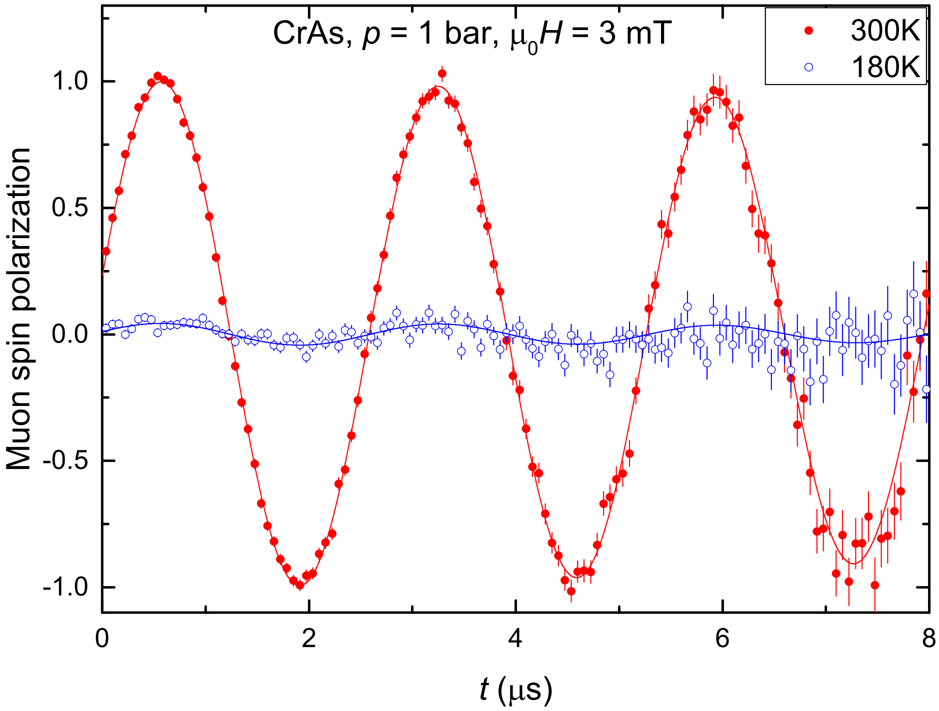

Figure 2. WTF μSR time-spectra ($\mu_0 H = 3$ mT) of CrAs measured below ($T = 180$ K) and above ($T = 300$ K) the magnetic transition transition temperature ($T_N \simeq 265$ K) at $p = 1$ bar.

Figure 2 represents the wTF μSR time spectra measured at ambient pressure above ($T \simeq 300$ K) and below ($T \simeq 180$ K) the magnetic transition ($T_N \simeq 265$ K). The solid lines correspond to the fit of the first term on the right-hand side of Eq. (4) to the experimental data. The "magnetic term" $[A_m(0)P_{ZF}(t)]$ vanishes within the first ~ 0.1 μs and thus is not observed with the present data binning ($\simeq 0.063$ μs). The "pressure cell" contribution is missing since experiments under ambient pressure were performed by using the sample outside of the cell on the low-background GPS spectrometer.

Figure 3 demonstrates the dependence of the non-magnetic volume fraction $f = A_{nm}(0)/[A_{nm}(0) + A_m(0)]$ on temperature at various pressures.

TF μSR experiments

Figure 4 shows the TF μSR time spectra measured at $T = 0.24$ K and 1.5 K at $p = 5.8$ kbar. The stronger damping at $T = 0.24$ K is due to inhomogeneous field distribution caused by formation of the flux line lattice (FLL) in the superconducting CrAs.

The TF μSR data were analyzed by using the following functional form:

$$A(0)P(t) = A_{nm}(0)\, e^{-[\sigma_{nm}^2 + \sigma_{sc}^2]t^2/2} \cos(\gamma B t + \phi) + A_m(0) P_{ZF}(t) + A_{pc}(0) P_{pc}(t). \quad (5)$$

Here $A_{nm}(0)$, A_m, ϕ, and σ_{nm} have similar meanings as in Eq. (4), σ_{sc} is the relaxation rate caused by FLL formation, and B is the magnetic field inside the sample. Due to the diamagnetism of the superconducting state $B < B_{ex}$ for $T < T_c$ and $B \simeq B_{ex}$ for $T \geq T_c$.

Comparison of $T_c(p)$ from the present study with the literature data

Wu *et al.*[S6] have determined the transition temperature T_c as the temperature where the resistivity reaches their close to zero value. The such determined T_c continuously increases (from $\simeq 0.5$ K to $\simeq 1.5$ K) in the region where the superconductivity and magnetism coexists, reaches their maximum value ($\simeq 1.5$ K) at $p \simeq 11$ kBar and further decreases with increasing pressure [see Fig. 5(a)]. The onset temperature of superconducting transition (T_c^{onset}), which could be determined as the temperature

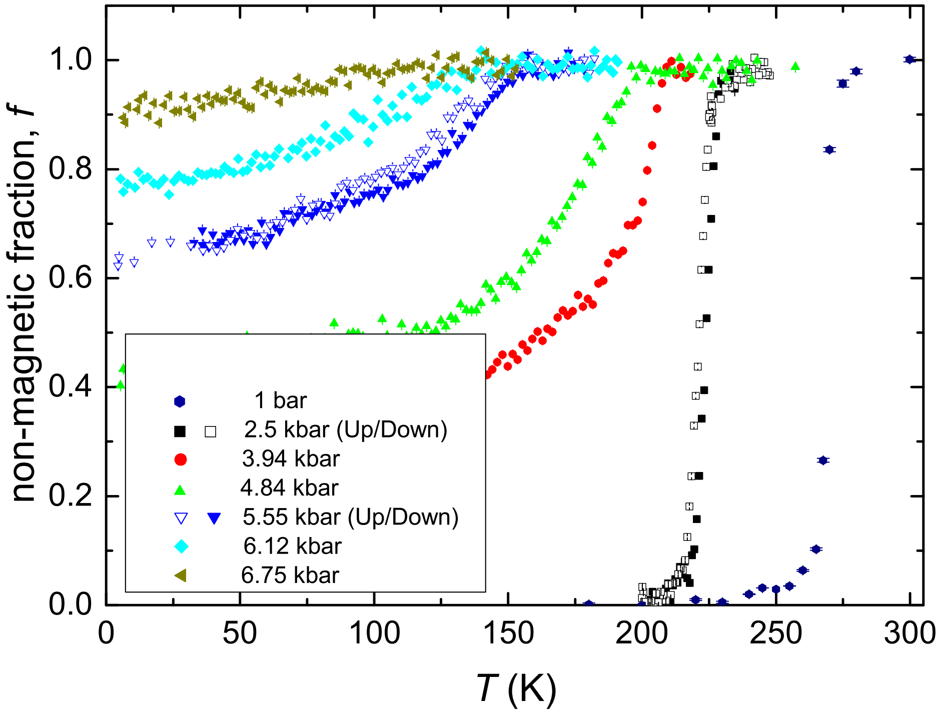

Figure 3. Temperature evolution of the non-magnetic volume fraction $f = A_{nm}(0)/[A_{nm}(0)+A_{m}(0)]$ of CrAs obtained in wTF μSR measurements at $p = 1$ bar, 2.5, 3.94, 4.84, 5.55, 6.12, and 6.75 kbar. Closed and open symbols correspond to the experimental data obtained with increasing and decreasing temperature, respectively.

where the resistivity curve starts to deviate from the straight line, behaves differently [see Fig. 5(a)]. Obviously, the two-step transition as observed by Wu *et al.* in the intermediate pressure range (Fig. 1b in Ref. 1) suggest the separation of the sample on the magnetic and the superconducting domains. Consequently, the T_c^{onset} would correspond to appearance of superconducting domains with relatively high transition temperature, while T_c is determined by occurrence of Josephson type coupling between the superconducting domains.

The comparison of $T_c(p)$ data obtained within the present study with T_c^{onset} from Ref.[S6] is shown in Fig. 5(b) and results in fare good agreements between two sets of the data. Note that the transition temperatures in Fig. 5(b) are normalized to their values at $p \simeq 10$ kbar.

References

S1. Andreica, D. Magnetic phase diagram in some Kondo-lattice compounds. Ph.D. thesis, ETH-Zurich (2001).

S2. Keller, L. *et al.* Pressure dependence of the magnetic order in CrAs: A neutron diffraction investigation. *Phys. Rev. B* **91**, 020409(R) (2015).

S3. Schenck, A., Andreica, D., Gygax, F.N. & Ott, H.R. Extreme quantum behavior of positive muons in $CeAl_2$ below 1 K. *Phys. Rev. B* **65**, 024444 (2001).

S4. Yaouanc, A. & Dalmas de Réotier, P. *Muon Spin Rotation, Relaxation and Resonance: Applications to Condensed Matter* (Oxford University Press, Oxford, 2011).

S5. http://jick.net/ jess/ppt/GardenExport/node9.html.

S6. Wu, W. *et al.* Superconductitivy in the vicinity of the antiferromagnetic order in CrAs. *Nat. Commun.* **5**, 5508 (2014).

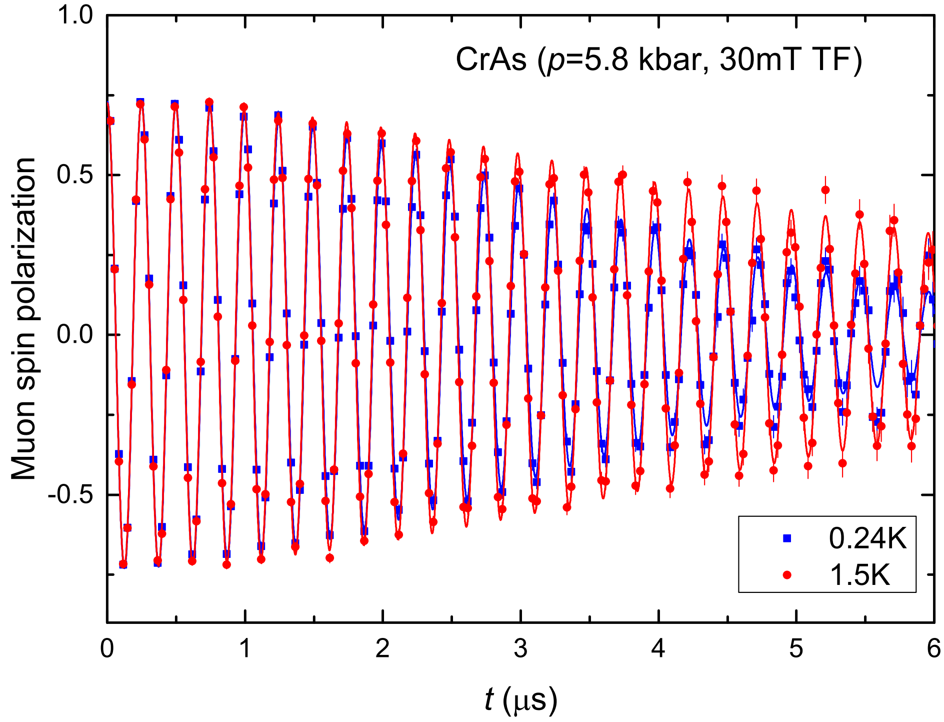

Figure 4. TF μSR time-spectra ($\mu_0 H = 30$ mT) of CrAs measured below ($T = 0.24$ K) and above ($T = 1.5$ K) the superconducting transition temperature ($T_c \simeq 1.1$ K) at $p = 5.8$ kbar. The stronger damping in the superconducting state is due to the formation of the flux line lattice.

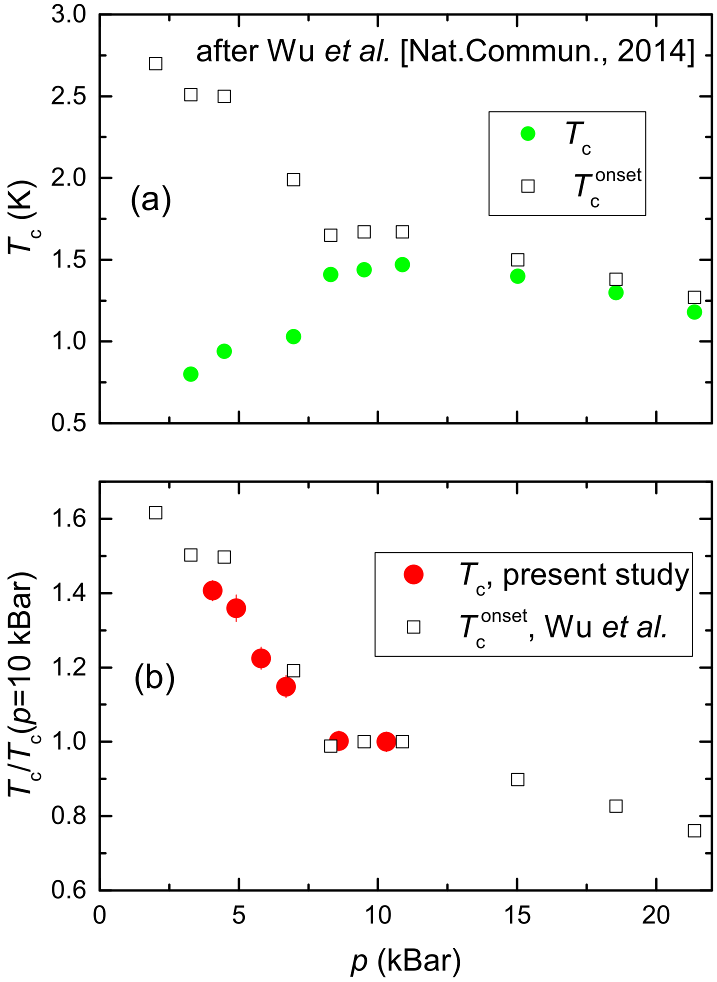

Figure 5. (a) T_c and T_c^{onset} as determined from the resistivity data of Wu *et al.* [see Figs. 1(b) and (c) in Nat. Commun. **5**, 5508 (2014)]. (b) Comparison of $T_c(p)$ data obtained within the present study with $T_c^{onset}(p)$ after Wu *et al.* [Nat. Commun. **5**, 5508 (2014)].
